# Dynamics of investor spanning trees around dot-com bubble

**DOI:** 10.1371/journal.pone.0198807

**Published:** 2018-06-13

**Authors:** Sindhuja Ranganathan, Mikko Kivelä, Juho Kanniainen

**Affiliations:** 1 Laboratory of Industrial and Information Management/Tampere University of Technology, Tampere, Finland; 2 Department of Computer Science, School of Science/Aalto University, Espoo, Finland; Central European University, HUNGARY

## Abstract

We identify temporal investor networks for Nokia stock by constructing networks from correlations between investor-specific net-volumes and analyze changes in the networks around dot-com bubble. The analysis is conducted separately for households, financial, and non-financial institutions. Our results indicate that spanning tree measures for households reflected the boom and crisis: the maximum spanning tree measures had a clear upward tendency in the bull markets when the bubble was building up, and, even more importantly, the minimum spanning tree measures pre-reacted the burst of the bubble. At the same time, we find less clear reactions in the minimal and maximal spanning trees of non-financial and financial institutions around the bubble, which suggests that household investors can have a greater herding tendency around bubbles.

## Introduction

The strategic interaction and collection of individuals or agents in a financial setup can play a key role in determining their financial outcomes. Understanding how investors behave and operate has been a topic of interest in behavioral finance in the recent past. Previously, investor trading strategies and investor behavior were studied at an aggregated level using conventional regression methodologies [1, 2, 3, 4, 5, 6, 7]. In addition, the evolution of networks of stocks and currency rates and their structural change have been successfully analyzed in the existing literature [8, 9, 10, 11, 12, 13]. The effects of the economic and financial bubbles on the stock market have also been analyzed in the literature [14, 15, 16]. However, *investor networks* have received much less attention, and even though complex network methods have been applied to identify investor networks [17, 18, 19, 20], research studying the dynamics of investor networks around a financial crisis is lacking. This paper aims to take the first step toward providing an understanding of investor networks by focusing on the dynamics of investor correlation networks during the dot-com (IT Millennium) bubble using unique investor transaction registry data, which contain all the trades of Finnish households and institutions in Helsinki Exchange. In particular, we focus on the question of how gradual and non-gradual changes in investor network structures are related to the stock price process. This research opens avenues to increase the understanding of the actual mechanisms of stock markets to identify domino effects that can propagate through investors and propel the stock markets into a crisis state.

In this paper, investor correlation matrices are obtained using time series of investor-specific daily net volumes for Nokia, a major Finnish technology company, around the millennium. At the same time, Nokia is the most liquid stock in our data sample from the Helsinki stock exchange, and there has been other research based on the company’s stock market data, for example, by [18, 21, 22]. Investors’ correlation matrices are estimated for three main categories of investors: financial institutions, households, and non-financial institutions. Correlation matrices can be interpreted as link-weighted networks, and the links in the resulting networks where all nodes are connected can be filtered with a multitude of different approaches [12, 23, 24, 25]. An elegant and popular method in stock market network analysis is to employ minimal or maximal spanning tree methods to find a “backbone” of the full correlation network [8, 9, 11, 26, 27, 28]. Several more complicated correlation matrix construction and filtering methods have been developed recently [23, 25, 29, 30, 31, 32, 33], but utilizing these methods is left for future research.

The analysis of investor networks dynamics in this paper introduces two theoretical challenges when compared to other financial correlation networks. First, the set of investors is much larger than, for example, the number of stocks, and the set of active investors is strongly time-varying. The vast majority of methods developed for analyzing dynamic, or temporal, networks are based on the assumption that only the links change while the set of nodes is stable [34, 35]. Further, changes in the set of investors limits the applicability of methods based on analyzing each network snapshot separately, as metrics that are sensitive to network size cannot be compared across different time windows, where the number of investors can vary significantly. The second challenge is related to the widely varying sparsity of the time series, where few investors are extremely active and many others trade very infrequently. The active investors could be investigated using high temporal resolution and short observation window lengths, but the infrequent investors must be examined using lower resolution and a longer time window. The conventional correlation analysis performed here requires that a single time resolution level and observation window length be chosen, and this choice must be a compromise between the two extremes.

We construct minimum and maximum spanning trees for networks within six-month time windows, with a displacement of one month. Our results with estimated correlations between households’ transactions show that the average weight of maximum spanning tree increases and the average weight of minimum spanning tree decreases before the tipping point of the stock prices (at which stock prices start to decline), after which they remain quite stable. In other words, when the bubble propagates, on average, an investor has more positive correlations with other investors in the maximum spanning tree. At the same time, however, the correlations with the most distant investor, in terms of trading style, become even more negative in the minimum spanning tree. This suggests that households became polarized before the Nokia price crash in 2000. However, as no strong effect can be observed for financial institutions the average weights of the minimum and maximum spanning trees of institutional investors are not as clearly related to the evolution of the financial crisis.

### Dot-com bubble

In this paper, we analyze the behavior of Nokia’s investors around the dot-com bubble in 2000. Bubbles are a phenomenon where the prices of assets deviate from their fundamental values [36]. Generally, during bubbles, investors purchase shares anticipating future gains. When the bubbles collapse, there is a sudden fall in prices, and that was the case in the dot-com bubble. Particularly, during the late 1990s, internet-based stocks dominated the equity markets, and there was heavy investment in the internet and technology-based start-ups with extremely optimistic expectations. As investors started pouring money into technology-based start-up companies, the prices of the companies’ shares grew very high. Then, in early 2000, investments in these companies reduced drastically, and many of these companies that were expected to generate profits failed, leading to the bursting of the bubble. Consequently, there was panic selling and the market slumped.

Bubbles have been studied quite extensively and from various perspectives. According to [37], market prices during bubbles follow power-law acceleration and have log-periodic oscillations. The dot-com bubble had similar characteristics and resulted in a crash (see, for example, ref. [38]). One perspective is that bubbles occur due to the uncertainty that prevails in the market [39]. In this regard, ref. [40] provides evidence that uncertainty is a plausible cause for a sudden rise in the price of some stocks. Similarly, the high level of uncertainty matched the high prices and high return volatility in the market during the dot-com bubble. The sudden rise and fall in market prices during the dot-com bubble was associated with variations in risks from various sources. Bakshi and Wu [41] show that with the rising valuation of the NASDAQ 100, return volatility as a risk measure increased, estimates for the market price for diffusion risk became negative (from September 21, 1999 to January 5, 2000), and the market price of jump risk became unusually high. Another perspective on bubbles is that they occur when there are new innovations [42] that investors see as opportunity pulls, anticipating high profits in the future. Other reasons for the occurrence of a bubble are a lack of experience on the part of traders [43], investor’s emotions [44], investor over-confidence [45], and public announcements [46]. There are several reasons for a bubble to burst. According to [40], one of the reasons that the dot-com bubble burst was that the expected profitability of technology stocks became low. Not all bubbles lead to crashes, but when a bubble does crash, it signals important information to the market. According to [42], a burst signals that there is a need to implement new innovations that occurred during the bubble period. This requires social and economic support to continue the growth of innovations that could benefit the economy.

## Results

Next, we describe how we construct a series of correlation networks of investors investing in Nokia stock around the dot-com bubble (1998–2002) and report the basic statistics related to the changes in these networks. We then continue to investigate the minimal and maximal spanning trees we extract from these fully connected networks. We report the results of our analysis separately for Finnish households, financial institutions, and non-financial institutions.

### Nodes in the networks: Active investors

**Fig 1:**
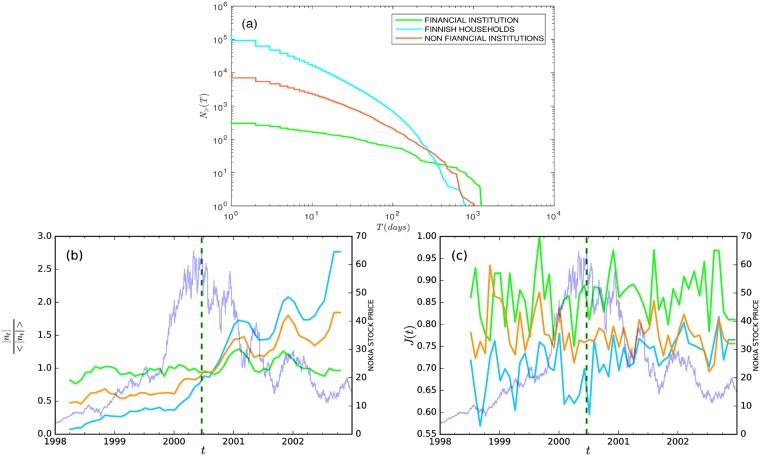
The number of investors in Nokia stocks during the period 1998–2002 and the change in the number of investors across the six-month time windows. (a) The number of investors *N*_>_(*T*) who traded the Nokia stock at least on *T* different days during the whole time period 1998–2002 (i.e., a non-normalized complementary cumulative distribution). (b) The evolution of the number of investors trading Nokia in the six-month time windows for households, non-financial institutions, and financial institutions. The numbers of investors in each category |*n*_*t*_| vary widely across categories, and they are normalized by the average numbers of investors in the full time period 〈|*n*_*t*_|〉. (c) The change in the number of investors is measured using the Jaccard coefficient for different investor categories. The value of *J*(*t*) is higher (lower) the more (less) similar the consecutive networks are in terms of nodes in them (see Eq 1). Results for each time window in panels (b) and (c) are plotted at the end of the window. That is, each point is estimated with data over the previous 126 trading days (6 months). The estimation windows are rolling by one month, and the resulting points are joined by solid lines. In panels (b) and (c), the green dotted vertical line in the figures represents the highest stock price of Nokia in the sample period, and the blue curves (with axis on the right) represent the Nokia stock price. In all panels, the lime-green curve corresponds to financial institutions, the cyan curve to households, and the orange curve to non-financial institutions.

Investors form the nodes of the network we construct, and to estimate the correlations between pairs of them, we need to have enough data on their trading behavior. Fig 1a depicts the distributions of investors divided into three categories in the period 1998–2002. Many investors traded for only a few days but relatively few traded for many days, making the data sparse. We take two steps to alleviate the problems related to sparse data in the network construction: First, we only consider *active investors* who have traded for a minimum of 20 days in a given time period. Second, daily net volumes of each active investor are averaged over a week (that is, we apply investor-specific simple moving averages).

We investigate our total sample period 1998–2002 with six-month sliding time windows using a one-month rolling window on it. Using the above definition for active investors for each six-month time window, Fig 1b depicts the evolution of the number of active financial institutions, households, and non-financial institutions within these time windows. We see that the numbers of active households and non-financial institutions showed positive trends over the sample period, while the number of active financial institutions remained rather stable. Importantly, the bubble “burst” did not have clear effects on the number of active traders.

Even when the number of investors in each time window is relatively stable, the set of investors can vary significantly. This is indeed the case, as shown by Fig 1c which displays the number of investors overlapping in every subsequent time window measured by the Jaccard index defined as:

$$J\left(t\right)=\frac{|n_{t+1}\cap \;n_{t}|}{|n_{t+1}\cup \;n_{t}|}\;,$$ (1)

where *n*_*t*_ and *n*_*t*+1_ represent the sets of nodes in the networks with time windows ending in months *t* and *t* + 1. For example, for households, the Jaccard index can get values as low as 0.6, which means that 40% of investors in two subsequent time windows were only present in one of them. To put this percentage into perspective, it is worth noting that out of the total of seven months spanned by the two windows, there is a five-month overlap. That is, one could expect a high Jaccard index even if there are major changes in the sets of investors. Note that the activeness criterion (at least 20 observations in six months) is applied for each estimation period with a displacement of one month, and this filtering has an effect on the Jaccard index values. We observe that the networks of households have lower similarity to each other compared to financial institutions, meaning that the turnover of active household investors is relatively high over time. Thus, especially for the relatively inactive household investors, the networks in different time windows are bound to be very different, and any stability observed in network statics cannot be solely explained by the stability of the networks; other organizing principles in the system also have an effect.

### Links in the networks: Correlations in trading patterns

We use the Pearson correlation of trading patterns of investor pairs inside each time window to construct links between the investors (for details, see Methods). The Pearson correlation coefficient has been used extensively in the network analysis of time series of stock prices [8], and it also has some clear advantages in the analysis of individual investor trading. Observations of exceptionally high trading volumes can represent days on which important information has arrived. It is of interest to analyze whether investors react to these information in the same way, and therefore it is desirable that the measure is sensitive to exceptionally large values. In contrast to the Pearson correlation, Kendall and Spearman correlations consider rank-order as opposed to metric information, and thus they do not weight these outlier days appropriately.

**Fig 2:**
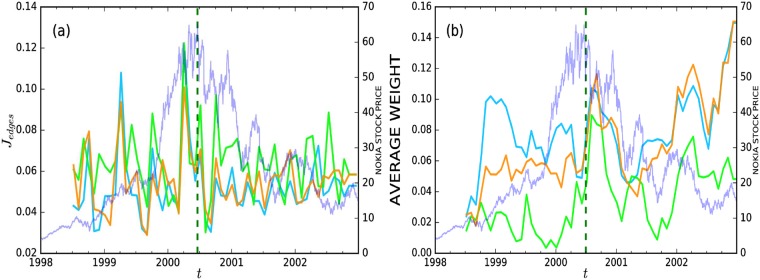
The change in investor correlations of Nokia stock trading across the six-month time windows during 1998–2002. (a) The average change in correlations between two consecutive time windows *J*_*edges*_(*t*) (see Eq 2 in the Methods section). (b) The average edge weight, or correlation, in each time window. The green dotted vertical line represents the highest stock price of Nokia in the sample period, and the blue curves (with axis on the right) represent the Nokia stock price. The lime-green curves correspond to financial institutions, the cyan curves to households, and the orange curves to non-financial institutions.

The nodes change between the different time windows, and the weights of the links (the correlations) are also relatively unstable. To quantify this, we show the average absolute change in correlations between nodes that remain in two consecutive time windows (see Eq 2 in Methods) and the average correlation between all pairs of nodes in Fig 2. The change in correlations between two consecutive time windows is on average lower (0.04–0.12) than the standard deviation of the correlations inside the time windows (0.14–0.23), but it is still very clearly within the same order of magnitude. That is, the network is relatively unstable in its links, but, as we will see below, the global organization of the network and related statistics are still rather stable.

### Minimum/Maximum spanning trees

The correlation matrices of investors’ net trading volumes can be interpreted as weighed networks where all node pairs (i.e., investors) are connected. Specifically, investors *i* and *j* are connected by a weight of *ρ*_*ij*_ ∈ [−1, 1], which is the Pearson correlation coefficient between investors’ daily net volumes. Clearly, the topological structure of these fully connected graphs is trivial, and all the information is in the weights. To analyze the structure, one needs to filter out parts of the edges, and there are various approaches for doing so [23, 24, 25, 33]. Following the literature on the analysis of stock prices [8, 9], we employ one of the simplest filtering methods and construct maximum (and minimum) spanning trees of correlation networks.

The idea of maximum spanning tree analysis is to filter out as many edges as possible so that the network is still connected and the highest possible weights (or, correlations) are not filtered out (for details, see the Methods section). According to ref. [17], information links may be identified from realized trades, and thus traders identified with similar trading behavior can have an (private) information channel. In light of this idea about the inference of information transfer in investor networks, the maximum spanning tree would reflect the smallest set of interactions that connect all investors and still have the strongest information flow between them. The interpretation of the empirical investor network as the information network, however, can be questioned, as two investors could certainly trade in the same directions without even knowing each other if they just follow the same investment strategies with the same public information channels. Generally speaking, the maximum spanning tree picks the most similar trading strategies while keeping the graph connected, whether or not it reflects the actual information channels. The average weight of maximum spanning tree shows how investors, on average, are pulled together or dispersed in a connected graph, and this quantity has been previously shown to react to crisis in stock price correlations [9]. The minimum spanning tree, on the other hand, reflects distant trading strategies, and its average weight can be used to analyze divergent trading strategies in a connected graph of investors. Particularly, with the minimum spanning trees, we investigate low, or even negative, correlations between investors’ net volumes. Conversely, the negative correlations reflect the fact that investors net volumes are negatively related, and thus they indicate divergent trading.

**Fig 3:**
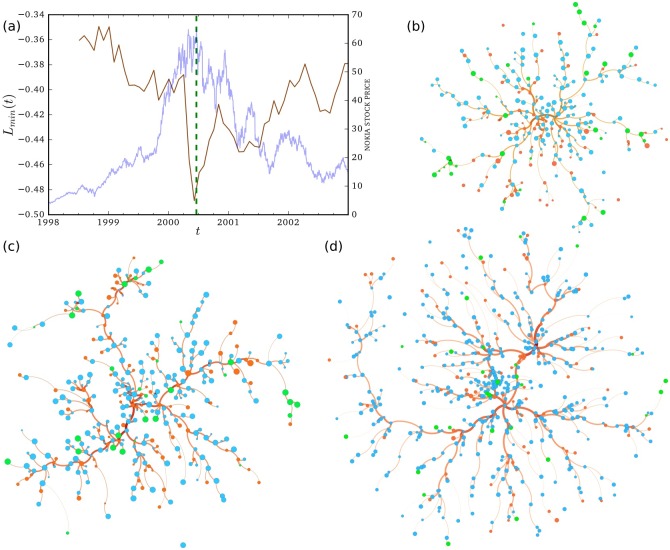
The minimum and maximum spanning trees of all investors. (a) Backward looking average weight of the minimum spanning tree, *L*_*min*_(*t*), for the merged set of investors with six-month time windows during 1998–2002 (brown line). The green dotted vertical line in the figures represents the highest stock price of Nokia in the sample period, and the blue curves (with axis on the right) represent the Nokia stock price. Maximum spanning trees between (b) July 8, 1999 and January 4, 2000 (before the crisis), (c) January 5, 2000 and July 6, 2000 (during the crisis), and (d) July 7, 2000 and January 4, 2001 (after the crisis). The cyan nodes represent households, the orange nodes non-financial institutions, and the lime-green nodes financial institutions. The sizes of the nodes are based on the volume traded by the investor during the period. However, one should not compare the sizes of nodes between different networks, as the sizes are not comparable across panels.

Fig 3a shows the evolution of the average weight of the minimum spanning tree, *L*_*min*_, for the *merged* network of investors in the three categories. There is an obvious, downward jump in *L*_*min*_ just before the tipping point, which is defined as the highest price of the Nokia stock during the sample period. Importantly, *L*_*min*_ is estimated using data from the past, and therefore no information about the forthcoming bubble burst was used. That is, the investors pre-reacted to the impending decline in the stock price. Next, we focus on investigating which investor groups are behind this reaction. We visualize the maximum spanning trees in Fig 3b, 3c and 3d. There does not seem to be any clearly visible clustering of categories similar to business sectors in stock networks or geographical regions in currency networks [8, 47, 48]. However, we can see that there might be some local tendency for nodes from the same category to be adjacent; however, this observation is not investigated further here.

**Fig 4:**
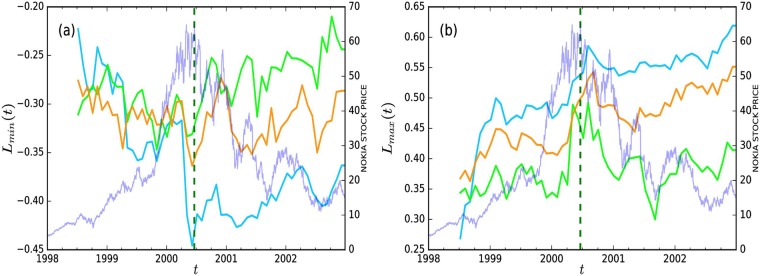
Backward looking average weight of the (a) minimum spanning tree, *L*_*min*_(*t*), (b) maximum spanning tree, *L*_*max*_(*t*) for different investor categories with six-month time windows during 1998–2002. The green dotted vertical line in the figures represents the highest stock price of Nokia in the sample period. The lime-green curve corresponds to the plot for Finnish financial institutions, the cyan curve corresponds to the plot for Finnish households, and the orange curve corresponds to the plot for Finnish non-financial institutions.

Fig 4 displays the average weights of minimum and maximum spanning trees, *L*_*min*_ and *L*_*max*_, around the crisis for networks containing nodes only from one of the three investor categories. Again, every data point is estimated with data over the previous 126 trading days (six months), and the estimation windows are rolling by one month. Fig 4a shows that the average weight of the minimum spanning tree, *L*_*min*_, of the household network suddenly jumps down a few months prior the turning point of the stock price evolution around the crisis. Particularly, the value of *L*_*min*_ was -0.32 on April 3, 2000, whereas it was -0.45 on June 6, 2000, after which the stock prices started to burst. Importantly, the difference is considerably large in comparison to other changes in the data sample; however, the estimates, -0.32 and -0.45, are based on partially overlapping estimation data (the length of the estimation period is six months, and the analysis is run with a rolling window of one month). Another important observation is that the level of *L*_*min*_ does not recover back to its level as prior to the tipping point during the following two years. For non-financial and financial institutions, we see no obvious patterns in *L*_*min*_ around the crisis. Overall, weights in the minimum spanning trees among households are, on average, abnormally negative just around the turning point for households. This means that households, on average, have neighbors in the minimum spanning tree who are trading in an abnormally opposite way.

The dynamics of the maximum spanning trees in Fig 4b provide a slightly different story compared to the minimum spanning tree dynamics. In particular, we see that the average weight of the maximum spanning tree, *L*_*max*_, for households shows a clearly positive trend prior the spike of February 2000, after which it remains quite stable. Specifically, its value was 0.27 in 1998, increasing to almost 0.6 in two years in bull markets, which is an increase of *122%*. This means that there are investors that were trading more similarly when the bubble was building up. A positive pre-trend and rather stable post-trend can also be identified for non-financial institutions, although it is weaker compared to households. Financial institutions, however, behave differently regarding *L*_*max*_–there is a peak in *L*_*max*_ for financial institutions just before the tipping point, which lasts half a year, but otherwise *L*_*max*_ is relatively stable over the period. Note that the average weights of the networks displayed in Fig 2b do not display peaks at the same times or of the same magnitude.

In light of private information channels that investors use in trading in stock markets (see [17]), our maximum spanning tree analysis results suggest that household investors’ connections to their most important neighbors in the connected graph became increasingly important when the techno bubble was building up, which could indicate herding in the stock market Moreover, the existing literature provides evidence that spanning trees for different financial networks react around financial crises, although with different data sets (and thus with different networks) compared to the present research (see [9, 49] with data on stock returns, [13] with data on stock market indexes, and [50] with data on currency exchange rates).

## Discussion

This paper examines the behavior of Finnish investors using shareholding registration records for Nokia stock on the Helsinki stock exchange from 1998–2002, which includes the period of the dot-com bubble. Analyses for households, non-financial institutions, and financial institutions are conducted using minimum and maximum spanning trees constructed from correlations between investor-specific net-volumes. We find that the spanning tree measures reflected the bubble with the data for households, and, in fact, they pre-reacted to forthcoming bear markets, whereas non-financial and financial institutions show no equally clear reactions. In particular, the average correlations of households’ minimum spanning trees clearly jumped down a couple of months before the Nokia price started to show a negative trend. Conversely, the average correlation in households’ maximum spanning tree dynamics did not jump suddenly right before the burst of the bubble—rather, the average correlation had a considerably large upward trend in bull markets, increasing from 0.27 to almost 0.60 in the two years before the stock price crash, after which it remained quite stable. The analysis was also conducted with 12-month rolling windows, and the results were the same (the results for this robustness check are available upon request). This result on maximum spanning trees could reflect information channels between individual household investors—investors’ connections to their most important neighbors in the connected graph becoming increasingly important when the techno bubble was building up, which could indicate herding in stock markets, especially among household investors. Based on our results, it could be argued that households were mainly responsible for the bubble and its burst. This question, however, needs more research with alternative approaches. For example, agent-based models estimated with actual transaction data could be used to elaborate the role of households in more detail.

There are some restrictions in our research on correlated investor networks, which are mainly related to how the networks are constructed. We used data on investors’ transactions with only one stock, as the other stocks in the our data set were too illiquid to have enough data estimating investor-specific networks. In future studies, multiple similar stocks could be pooled together or methods that function better with sparse data could be used. Another limitation is the way the Pearson correlation was used between the investment time series to calculate the similarities between nodes. There are more sophisticated ways of inferring the latent relationships between the nodes in the literature [29, 30, 31, 32], but the particular challenge in investor networks is the high variation in the transaction frequencies between investors. High-frequency nodes can be analyzed with much higher temporal resolution than low-frequency ones, and choosing a single resolution level involves a compromise between these two extremes. Finally, the spanning tree analysis discards valuable data in a very aggressive way to make the system less complex, and there are multiple alternatives in the literature where more data is kept [23, 24, 25, 33]. In future research, we aim to build the network in a more sophisticated way, which will allow us to analyze a large number of stocks with alternative methods.

The network of investors is changing dynamically, and the approach taken here—which is in line with the literature on stock correlation networks—was intended to calculate various static network metrics on snapshots of the network and then inspect how those metrics change over time. Methods that do not rely on static networks but measure the dynamics of networks have been developed in the field of temporal networks [34, 35], but most of these approaches have been constructed for networks where the links change dynamically but the nodes are relatively stable. There are, of course, other systems with long temporal data and large changes in the set of nodes, such as citation networks and collaboration networks [51, 52, 53]. In some systems, such contact networks of customers, the patterns of nodes’ leaving and entering the system can even be of primary interest [54, 55, 56]. However, there are relatively few methods for analyzing networks in which both nodes and links change, and the temporal investor networks introduced here could serve as a good example for network analysis in future research. To faciliate this we have made the investor correlation matrices public (see the Methods section).

Additionally, in the present paper, the set of investors was organized based on the status of household, financial institution, or non-financial institution and activeness, which is a rather arbitrary way to classify investors. Also, one could argue that the observations of investor trading events are just realizations of a non-observable (psychological) process, making the identified temporal network unstable. In our future research, we will develop sampling methods to overcome these potential problems. In addition, filtering and community-detection methods [57] as well as alternative inference techniques for the estimation of network edges are expected in our future research.

## Materials and methods

### Data

The data used in this study come from the central register of shareholdings for Finnish stocks from the Finnish central depository, provided by Euroclear Finland. The data set includes all the major publicly traded Finnish stocks from 1995. It consists of shareholdings of all Finnish and non-Finnish investors traded in the Helsinki stock exchange on a daily-level basis. The data contain investors’ trades and portfolios, including all Finnish household investors, Finnish institutions, and foreign institutions. The records are exact duplicates of the official certificates of ownership and trades, and hence they are very reliable. The Book Entry System entails compulsory registration of holdings for Finnish individuals (referred to as households) and institutions. Foreigners are partially exempt from registration, as they can opt for registration in a nominee name, and thus they cannot be separated from each other. Thus, data about foreigners’ trades is excluded in the present paper. A more detailed descriptions of the data set is provide in [1, 18].

Our sample data consist of marketplace transactions of **Nokia** stock, consisting of investor transactions from January 1, 1998 to December 2002. Each data record contains the following information: stock ticker, owner id, trading date, transaction registration date, number of shares traded, the price of trade, buy/sell transaction type, and other investor-specific fields, such as investors’ sector code, language code, gender, date of birth, and postal code. We have considered investors from different categories who have traded actively with Nokia in our analysis. Information about having the status of household, financial institution or non-financial institution was directly available from the data provider. Each investor has a unique investor ID, and for each ID, certain attributes are assigned, such as category. This information is self-provided by identifiable investors.

### Links in the network

Net volume traded by an investor *i* on day *t* is given as $V_{i,t}=V_{i,t}^{b}-V_{i,t}^{s}$, where $V_{i,t}^{b}$ is the number of Nokia shares bought by investor *i* on day *t*, and $V_{i,t}^{s}$ is the number of Nokia shares sold by investor *i* on day *t*. In comparison to the inference method introduced in [18], we do not scale the net volumes by $V_{i,t}^{b}+V_{i,t}^{s}$, as the scaled approach does not measure the magnitude of trades; that is, the level of the scaled variable does not reflect exceptionally high or low traded net volumes. For example, suppose that on a given day for a given stock, investor A buys one share and sells zero while investor B buys exceptionally many shares, say, 1,000,000 and sells zero. Then, both investors’ scaled net volumes would equal +1, although their trading behavior has been very different. The dependency between two investors, *i* and *j*, is measured with the Pearson correlation for *M* different time windows of fixed width *W*. In our study, *W* is set to 126 trading days (six months), and the analysis is run with six-month sliding time windows using a one-month (21 trading days) rolling window. As the total number of days in our data is 1252, these choices give us *M* = 54 time windows for the overall six-month time window.

Note that the data studied here are very sparse in the sense that, for many investors, most days are without any activity (see Fig 1a), although these silent days are here considered as intentional decisions not to trade. That is, the inactive days are not considered as missing data in our calculation of the Pearson correlation coefficient. In our notation, $\rho_{t}^{\left(ij\right)}$ denotes the Pearson correlation coefficient between investors *i* and *j* estimated from daily net volumes of *W* days, counted backwards from the day *t*. That is, the correlations between nodes at time *t* are defined as,

$$\rho_{t}^{\left(ij\right)}=\frac{\text{Cov}(\vec{V_{i,t}},\vec{V_{j,t}})}{\sqrt{\text{Var}\left(\vec{V_{i,t}}\right)\text{Var}\left(\vec{V_{j,t}}\right)}},$$

where $\vec{V_{i,t}}={\left\{V_{i,\tau }\right\}}_{\tau =t-W}^{t}$. One could also use the daily net volumes of W/2 days in the past and W/2 days in the future, but we prefer to use the data in the past instead of using the data in the future in order to analyze pre-reactions in the networks so that no information about the forthcoming bubble burst is unused.

The average absolute change in correlations between nodes that remain in two consecutive time windows is defined as

$$J_{edges}\left(t\right)=\frac{1}{|e_{t}\cap e_{t+1}|}\sum_{\left(i,j\right)\in e_{t}\cap e_{t+1}}(|\rho_{t+1}^{\left(ij\right)}-\rho_{t}^{\left(ij\right)}|),$$ (2)

where *e*_*t*_ denotes the set of edges in the network at time *t* (i.e., all pairs of nodes *e*_*t*_ = {(*u*, *v*)|*u*, *v* ∈ *n*_*t*_, *u* ≠ *v*} where *n*_*t*_ represent the sets of nodes in the network where the time window ends at month *t*). Data about correlations between investor pairs is available at https://doi.org/10.5061/dryad.5b8n621.

### Minimum and maximum spanning trees

For a network with |*n*_*t*_| nodes and edge set *e*_*t*_, a maximum spanning tree is a connected sub-network with the same nodes and a subset of |*n*_*t*_| − 1 edges $e_{t}^{max}\subseteq e_{t}$ such that the sum of the edge weights (here correlations), $\sum_{\left(i,j\right)\in e_{t}^{max}}\rho_{t}^{\left(ij\right)}$, is maximized. Similarly, for a minimal spanning tree, we find a set of edges $e_{t}^{min}$ such that the sum of the edge weights is minimized.

Note that we do not transform the correlations into distance using $d_{ij}=\sqrt{2(1-\rho_{t})}$, which would turn minimal spanning trees into maximal ones and vice-versa—spanning tree structure is otherwise invariant to this transformation because this transformation only reverses the rank-order of the edge weights. We also construct minimum spanning trees, which are complementary to the maximum ones.

The average weights of maximum and minimum spanning trees are defined as:

$$L_{max}\left(t\right)=\frac{1}{\left(N_{t}-1\right)}\sum_{\left(i,j\right)\in e_{t}^{max}}\rho_{t}^{\left(ij\right)}.$$

and

$$L_{min}\left(t\right)=\frac{1}{\left(N_{t}-1\right)}\sum_{\left(i,j\right)\in e_{t}^{min}}\rho_{t}^{\left(ij\right)},$$

respectively.

The spanning trees were computed using Kruskal’s algorithm, which is a standard algorithm for finding minimum and maximum spanning trees for graphs that have positive weights. We used NetPython (available from https://github.com/CxAalto/netpython) network analysis software for computations.
